# Supplementary material for: Sarc-F and muscle function in community dwelling adults with aged care service needs: baseline and post-training relationship
Source: PeerJ. 2019 Nov 27;7:e8140. doi: 10.7717/peerj.8140 (PMC6884990; doi:10.7717/peerj.8140)
Supplement: Figure S1 — The predicted 1 repetition maximum (1RM) was a conservatively assumed based on the participant’s individual exercise ability and has been safely and effectively employed in similar populations (Henwood et al. 2008; Hewit et al., 2014). Exercise resistance was increased when participants were able to perform 12 repetitions for all 3 sets using the appropriate technique (Henwood et al., 2008). [file peerj-07-8140-s001.pdf]

Supplementary Figure 1: MUAD Progressive Resistance and Balance training protocol and programme.

	Conditioning phase		Training
	Wk 1 & 2	Wk 3 & 4	Wk 5 - 24
**Progressive Resistance training**			
1. Chest press<br>2. Seated row<br>3. Leg press<br>4. Leg curl<br>5. Leg extension<br>6. Leg adduction<br>7. Leg adduction<br>8. Abdominal crunch	2 sets,<br>8 repetitions @<br>50% predicted<br>1RM[1]	3 sets,<br>8 repetitions @<br>60% predicted<br>1RM	3 sets,<br>8-12 repetitions [2]<br>@ 75% predicted<br>1RM
**Balance exercises**			
1. Single leg stand	Increase stable surface/object support.<br>Reduces sets and repetitions/time.	Reduced support.<br>Increased repetitions/time	2 sets @ 20 s/leg
2. Tight rope walking			2 sets,<br>10 steps forward<br>& 10 back
3. Box stepping			5 X clockwise & 5 X anticlockwise
4. Calf raises			2 sets,<br>10 repetitions

Notes:

1. The predicted 1 repetition maximum (1RM) was a conservatively assumed based on the participant’s individual exercise ability and has been safely and effectively employed in similar populations (Henwood et al. 2008; Hewitt et al. 2014).
2. Exercise resistance was increased when participants were able to perform 12 repetitions for all 3 sets using the appropriate technique (Henwood et al. 2008).
