# Supplementary material for: Sarc-F and muscle function in community dwelling adults with aged care service needs: baseline and post-training relationship
Source: PeerJ. 2019 Nov 27;7:e8140. doi: 10.7717/peerj.8140 (PMC6884990; doi:10.7717/peerj.8140)
Supplement: Supplemental Information 3 [file peerj-07-8140-s003.docx]

library(FactoMineR)

library(nnet)

data = read.csv("SARCFData.csv")

########################## Baseline Analysis #########################################

# SARC F Sum

SARCFSum = data$Slowness + data$AssistanceWalk + data$RisingChair + data$ClimbingStairs + data$Falls

objectivedf = data.frame(SARCFSum= SARCFSum,Chair=data$Chairstand1, Grip=data$Gripstrength1,

Lean=data$Leanmasspercent1, Isometric=data$Isometricstrength1)

# Correlations

cor.test(objectivedf$SARCFSum,objectivedf$Chair,use = "pairwise.complete.obs",method="pearson")

cor.test(objectivedf$SARCFSum,objectivedf$Grip,use = "pairwise.complete.obs",method="pearson")

cor.test(objectivedf$SARCFSum,objectivedf$Isometric,use = "pairwise.complete.obs",method="pearson")

data$Slowness = factor(data$Slowness,levels=c(0,1,2), labels=c("Positive", "INT", "Negative"))

data$AssistanceWalk = factor(data$AssistanceWalk,levels=c(0,1,2), labels=c("Positive", "INT", "Negative"))

data$RisingChair = factor(data$RisingChair,levels=c(0,1,2), labels=c("Positive", "INT", "Negative"))

data$ClimbingStairs = factor(data$ClimbingStairs,levels=c(0,1,2), labels=c("Positive", "INT", "Negative"))

data$Falls = factor(data$Falls,levels=c(0,1,2), labels=c("Positive", "INT", "Negative"))

data$SARCF = factor(data$SARCF, levels=c(0,1), labels=c("Non-Sarcopenic", "Sarcopenic"))

# Multinomial Models

basemnomdf = data.frame(Slowness=data$Slowness, AssistanceWalk=data$AssistanceWalk,RisingChair=data$RisingChair,

ClimbingStairs=data$ClimbingStairs,Falls=data$Falls,

Chair=data$Chairstand1/sd(data$Chairstand1,na.rm=T), Grip=data$Gripstrength1/sd(data$Gripstrength1,na.rm=T),

Lean=data$Leanmasspercent1/sd(data$Leanmasspercent1,na.rm=T), Isometric=data$Isometricstrength1/sd(data$Isometricstrength1,na.rm=T))

slow1base1Chair=multinom(Slowness~Chair,data=basemnomdf)

summary(slow1base1Chair)

slow1base1Chairaz = summary(slow1base1Chair)$coefficients/summary(slow1base1Chair)$standard.errors

slow1base1Chairap = (1 - pnorm(abs(slow1base1Chairaz), 0, 1)) * 2

exp(coef(slow1base1Chair))

slow1base1Grip=multinom(Slowness~Grip,data=basemnomdf)

summary(slow1base1Grip)

slow1base1Gripaz = summary(slow1base1Grip)$coefficients/summary(slow1base1Grip)$standard.errors

slow1base1Gripap = (1 - pnorm(abs(slow1base1Gripaz), 0, 1)) * 2

exp(coef(slow1base1Grip))

# Slowness

slow1base1Chair=multinom(Slowness~Chair,data=basemnomdf)

summary(slow1base1Chair)

slow1base1Chairaz = summary(slow1base1Chair)$coefficients/summary(slow1base1Chair)$standard.errors

slow1base1Chairap = (1 - pnorm(abs(slow1base1Chairaz), 0, 1)) * 2

exp(coef(slow1base1Chair))

slow1base1Grip=multinom(Slowness~Grip,data=basemnomdf)

summary(slow1base1Grip)

slow1base1Gripaz = summary(slow1base1Grip)$coefficients/summary(slow1base1Grip)$standard.errors

slow1base1Gripap = (1 - pnorm(abs(slow1base1Gripaz), 0, 1)) * 2

exp(coef(slow1base1Grip))

slow1base1Isometric=multinom(Slowness~Isometric,data=basemnomdf)

summary(slow1base1Isometric)

slow1base1Isometricaz = summary(slow1base1Isometric)$coefficients/summary(slow1base1Isometric)$standard.errors

slow1base1Isometricap = (1 - pnorm(abs(slow1base1Isometricaz), 0, 1)) * 2

exp(coef(slow1base1Isometric))

slow1base1Lean=multinom(Slowness~Lean,data=basemnomdf)

summary(slow1base1Lean)

slow1base1Leanaz = summary(slow1base1Lean)$coefficients/summary(slow1base1Lean)$standard.errors

slow1base1Leanap = (1 - pnorm(abs(slow1base1Leanaz), 0, 1)) * 2

exp(coef(slow1base1Lean))

slow1base1Walk=multinom(Slowness~Walk,data=ppdata.wsp2.c)

summary(slow1base1Walk)

slow1base1Walkaz = summary(slow1base1Walk)$coefficients/summary(slow1base1Walk)$standard.errors

slow1base1Walkap = (1 - pnorm(abs(slow1base1Walkaz), 0, 1)) * 2

exp(coef(slow1base1Walk))

# AssistanceWalk

AW1base1Chair=multinom(AssistanceWalk~Chair,data=basemnomdf)

summary(AW1base1Chair)

AW1base1Chairaz = summary(AW1base1Chair)$coefficients/summary(AW1base1Chair)$standard.errors

AW1base1Chairap = (1 - pnorm(abs(AW1base1Chairaz), 0, 1)) * 2

exp(coef(AW1base1Chair))

AW1base1Grip=multinom(AssistanceWalk~Grip,data=basemnomdf)

summary(AW1base1Grip)

AW1base1Gripaz = summary(AW1base1Grip)$coefficients/summary(AW1base1Grip)$standard.errors

AW1base1Gripap = (1 - pnorm(abs(AW1base1Gripaz), 0, 1)) * 2

exp(coef(AW1base1Grip))

AW1base1Isometric=multinom(AssistanceWalk~Isometric,data=basemnomdf)

summary(AW1base1Isometric)

AW1base1Isometricaz = summary(AW1base1Isometric)$coefficients/summary(AW1base1Isometric)$standard.errors

AW1base1Isometricap = (1 - pnorm(abs(AW1base1Isometricaz), 0, 1)) * 2

exp(coef(AW1base1Isometric))

AW1base1Lean=multinom(AssistanceWalk~Lean,data=basemnomdf)

summary(AW1base1Lean)

AW1base1Leanaz = summary(AW1base1Lean)$coefficients/summary(AW1base1Lean)$standard.errors

AW1base1Leanap = (1 - pnorm(abs(AW1base1Leanaz), 0, 1)) * 2

exp(coef(AW1base1Lean))

AW1base1Walk=multinom(AssistanceWalk~Walk,data=ppdata.wsp2.c)

summary(AW1base1Walk)

AW1base1Walkaz = summary(AW1base1Walk)$coefficients/summary(AW1base1Walk)$standard.errors

AW1base1Walkap = (1 - pnorm(abs(AW1base1Walkaz), 0, 1)) * 2

exp(coef(AW1base1Walk))

# Chair Sit to Stand

CHAIRPER1base1Chair=multinom(RisingChair~Chair,data=basemnomdf)

summary(CHAIRPER1base1Chair)

CHAIRPER1base1Chairaz = summary(CHAIRPER1base1Chair)$coefficients/summary(CHAIRPER1base1Chair)$standard.errors

CHAIRPER1base1Chairap = (1 - pnorm(abs(CHAIRPER1base1Chairaz), 0, 1)) * 2

exp(coef(CHAIRPER1base1Chair))

CHAIRPER1base1Grip=multinom(RisingChair~Grip,data=basemnomdf)

summary(CHAIRPER1base1Grip)

CHAIRPER1base1Gripaz = summary(CHAIRPER1base1Grip)$coefficients/summary(CHAIRPER1base1Grip)$standard.errors

CHAIRPER1base1Gripap = (1 - pnorm(abs(CHAIRPER1base1Gripaz), 0, 1)) * 2

exp(coef(CHAIRPER1base1Grip))

CHAIRPER1base1Isometric=multinom(RisingChair~Isometric,data=basemnomdf)

summary(CHAIRPER1base1Isometric)

CHAIRPER1base1Isometricaz = summary(CHAIRPER1base1Isometric)$coefficients/summary(CHAIRPER1base1Isometric)$standard.errors

CHAIRPER1base1Isometricap = (1 - pnorm(abs(CHAIRPER1base1Isometricaz), 0, 1)) * 2

exp(coef(CHAIRPER1base1Isometric))

CHAIRPER1base1Lean=multinom(RisingChair~Lean,data=basemnomdf)

summary(CHAIRPER1base1Lean)

CHAIRPER1base1Leanaz = summary(CHAIRPER1base1Lean)$coefficients/summary(CHAIRPER1base1Lean)$standard.errors

CHAIRPER1base1Leanap = (1 - pnorm(abs(CHAIRPER1base1Leanaz), 0, 1)) * 2

exp(coef(CHAIRPER1base1Lean))

CHAIRPER1base1Walk=multinom(RisingChair~Walk,data=ppdata.wsp2.c)

summary(CHAIRPER1base1Walk)

CHAIRPER1base1Walkaz = summary(CHAIRPER1base1Walk)$coefficients/summary(CHAIRPER1base1Walk)$standard.errors

CHAIRPER1base1Walkap = (1 - pnorm(abs(CHAIRPER1base1Walkaz), 0, 1)) * 2

exp(coef(CHAIRPER1base1Walk))

# Stair Climbing

STAIR1base1Chair=multinom(ClimbingStairs~Chair,data=basemnomdf)

summary(STAIR1base1Chair)

STAIR1base1Chairaz = summary(STAIR1base1Chair)$coefficients/summary(STAIR1base1Chair)$standard.errors

STAIR1base1Chairap = (1 - pnorm(abs(STAIR1base1Chairaz), 0, 1)) * 2

exp(coef(STAIR1base1Chair))

STAIR1base1Grip=multinom(ClimbingStairs~Grip,data=basemnomdf)

summary(STAIR1base1Grip)

STAIR1base1Gripaz = summary(STAIR1base1Grip)$coefficients/summary(STAIR1base1Grip)$standard.errors

STAIR1base1Gripap = (1 - pnorm(abs(STAIR1base1Gripaz), 0, 1)) * 2

exp(coef(STAIR1base1Grip))

STAIR1base1Isometric=multinom(ClimbingStairs~Isometric,data=basemnomdf)

summary(STAIR1base1Isometric)

STAIR1base1Isometricaz = summary(STAIR1base1Isometric)$coefficients/summary(STAIR1base1Isometric)$standard.errors

STAIR1base1Isometricap = (1 - pnorm(abs(STAIR1base1Isometricaz), 0, 1)) * 2

exp(coef(STAIR1base1Isometric))

STAIR1base1Lean=multinom(ClimbingStairs~Lean,data=basemnomdf)

summary(STAIR1base1Lean)

STAIR1base1Leanaz = summary(STAIR1base1Lean)$coefficients/summary(STAIR1base1Lean)$standard.errors

STAIR1base1Leanap = (1 - pnorm(abs(STAIR1base1Leanaz), 0, 1)) * 2

exp(coef(STAIR1base1Lean))

STAIR1base1Walk=multinom(ClimbingStairs~Walk,data=ppdata.wsp2.c)

summary(STAIR1base1Walk)

STAIR1base1Walkaz = summary(STAIR1base1Walk)$coefficients/summary(STAIR1base1Walk)$standard.errors

STAIR1base1Walkap = (1 - pnorm(abs(STAIR1base1Walkaz), 0, 1)) * 2

exp(coef(STAIR1base1Walk))

# Falls

FALL1base1Chair=multinom(Falls~Chair,data=basemnomdf)

summary(FALL1base1Chair)

FALL1base1Chairaz = summary(FALL1base1Chair)$coefficients/summary(FALL1base1Chair)$standard.errors

FALL1base1Chairap = (1 - pnorm(abs(FALL1base1Chairaz), 0, 1)) * 2

exp(coef(FALL1base1Chair))

FALL1base1Grip=multinom(Falls~Grip,data=basemnomdf)

summary(FALL1base1Grip)

FALL1base1Gripaz = summary(FALL1base1Grip)$coefficients/summary(FALL1base1Grip)$standard.errors

FALL1base1Gripap = (1 - pnorm(abs(FALL1base1Gripaz), 0, 1)) * 2

exp(coef(FALL1base1Grip))

FALL1base1Isometric=multinom(Falls~Isometric,data=basemnomdf)

summary(FALL1base1Isometric)

FALL1base1Isometricaz = summary(FALL1base1Isometric)$coefficients/summary(FALL1base1Isometric)$standard.errors

FALL1base1Isometricap = (1 - pnorm(abs(FALL1base1Isometricaz), 0, 1)) * 2

exp(coef(FALL1base1Isometric))

FALL1base1Lean=multinom(Falls~Lean,data=basemnomdf)

summary(FALL1base1Lean)

FALL1base1Leanaz = summary(FALL1base1Lean)$coefficients/summary(FALL1base1Lean)$standard.errors

FALL1base1Leanap = (1 - pnorm(abs(FALL1base1Leanaz), 0, 1)) * 2

exp(coef(FALL1base1Lean))

FALL1base1Walk=multinom(Falls~Walk,data=ppdata.wsp2.c)

summary(FALL1base1Walk)

FALL1base1Walkaz = summary(FALL1base1Walk)$coefficients/summary(FALL1base1Walk)$standard.errors

FALL1base1Walkap = (1 - pnorm(abs(FALL1base1Walkaz), 0, 1)) * 2

exp(coef(FALL1base1Walk))

# MCA

sarcdata = data[,c(8:13)]

res.mca<-MCA(sarcdata,quali.sup=6, graph=FALSE)

res.mca$eig

plot(res.mca,invisible="ind", autoLab="y",cex=0.7, selectMod = "cos2 9")

plot(res.mca,invisible="ind", autoLab="y",cex=0.7, selectMod = "cos2 9",axes=c(1,3))

########################################################################
